# Vitamin D and Cardiovascular Disease, with Emphasis on Hypertension, Atherosclerosis, and Heart Failure

**DOI:** 10.3390/ijms21186483

**Published:** 2020-09-04

**Authors:** Nejla Latic, Reinhold G. Erben

**Affiliations:** Department. of Biomedical Sciences, University of Veterinary Medicine, 1210 Vienna, Austria; Nejla.Latic@vetmeduni.ac.at

**Keywords:** vitamin D, cardiovascular disease, hypertension, atherosclerosis, endothelial dysfunction, heart failure

## Abstract

Vitamin D deficiency is the most common nutritional deficiency, affecting almost one billion people worldwide. Vitamin D is mostly known for its role in intestinal calcium absorption and bone mineralization. However, the observation of seasonal changes in blood pressure and the subsequent identification of vitamin D receptor (VDR) and 1α-hydroxylase in cardiomyocytes, as well as endothelial and vascular smooth muscle cells, implicated a role of vitamin D in the cardiovascular system. Animal studies provided compelling evidence that vitamin D signaling is essential for cardiovascular integrity, especially for the regulation of vascular tone and as an antifibrotic and antihypertrophic signaling pathway in the heart. In addition, observational studies reported an association between vitamin D deficiency and risk of hypertension, atherosclerosis, and heart failure. However, recent clinical intervention studies failed to prove the causal relationship between vitamin D supplementation and beneficial effects on cardiovascular health. In this review, we aim to highlight our current understanding of the role of vitamin D in the cardiovascular system and to find potential explanations for the large discrepancies between the outcome of experimental studies and clinical intervention trials.

## 1. Introduction

Vitamin D is a secosteroid derived from cholesterol in animals (cholecalciferol, vitamin D_3_) and ergosterol in plants (ergocalciferol, vitamin D_2_). Although some studies reported that vitamin D_3_ and D_2_ are equipotent, there is accumulating evidence suggesting that vitamin D_3_ is more potent than vitamin D_2_ in raising total and free serum 25-hydroxyvitamin D (25OHD) levels in humans [1,2,3,4,5]. Both forms of vitamin D are referred to as vitamin D in this review. Vitamin D needs to be activated by 25-hydroxylation in the liver, yielding 25OHD, and by 1α-hydroxylation in the kidney, yielding the vitamin D hormone 1,25-dihydroxyvitamin D (1,25(OH)_2_D) [6]. It is currently thought that almost all biological actions of vitamin D are mediated by its active form, 1,25(OH)_2_D, signaling mainly through the intracellular vitamin D receptor (VDR) [6].

Seminal discoveries at the turn of the 20th century led to the identification of vitamin D deficiency as the cause of rickets [7]. Vitamin D deficiency is the most common nutritional deficiency, estimated to affect almost one billion people worldwide [8]. Multiple factors influence 25(OH)D levels, including nutrition, sunlight exposure, outdoor physical activity, and skin color. However, there is currently no consensus about defining optimal serum levels and dietary requirements. For example, the Endocrine Society defines deficiency as 25(OH)D levels below 50 nmol/L (20 ng/mL) but recommends at least levels above 75 nmol/L (30 ng/mL), and preferably between 100 and 150 nmol/L (40–60 ng/mL) [9], whereas other opinions recommend using the considerably lower cutoff of 12.5 ng/mL for the definition of vitamin D deficiency [10]. In addition, the threshold for sufficiency may be different for different diseases and conditions, making it difficult to set the optimal reference values.

For a very long time, it was thought that the only physiological role of vitamin D is the regulation of calcium and phosphate metabolism. However, about 40 years ago, Scragg et al. noticed a seasonality in patients suffering from cardiovascular disease and attributed it to the low 25OHD levels patients had in winter [11,12,13]. Studies investigating the potential benefits of vitamin D supplementation for cardiovascular health followed. Moreover, the discovery of VDR and 1α-hydroxylase in extra-osseous tissues indicated additional physiological roles of vitamin D, apart from its well-known role in mineral homeostasis. Epidemiological data indicate that vitamin D deficiency in humans is associated with arterial stiffness, hypertension, left-ventricular hypertrophy, and endothelial dysfunction in patients with chronic kidney disease, as well as in normal subjects, fostering the belief that vitamin D has a protective role in cardiovascular diseases, and that it may lower the risk for heart failure [14,15,16,17].

However, a large Mendelian randomization study did not confirm the association between vitamin D status and cardiovascular disease (CVD) [18], and the recently performed large intervention studies vitamin D and omega 3 trial (VITAL) and vitamin D assessment study (ViDA) failed to demonstrate a beneficial effect of vitamin D supplementation on cardiovascular outcomes [19,20,21]. Nonetheless, animal studies provided compelling evidence in favor of the important role of vitamin D signaling in the cardiovascular system.

The purpose of this review is to highlight our current understanding of the role of vitamin D in cardiovascular health, focusing on hypertension, atherosclerosis, and heart failure, and to find potential explanations for the large discrepancies between the outcome of experimental studies and clinical intervention trials. 

## 2. Vitamin D Metabolism

Vitamin D metabolism is a complex process involving multiple organs [6,22]. Vitamin D can be synthetized de novo in the skin by exposure to ultraviolet B (UVB) light that converts 7-dehydrocholesterol to cholecalciferol, or it can be taken up by ingestion. Cutaneously synthetized or ingested cholecalciferol binds to the vitamin D-binding protein and is transferred to the liver. The hepatic enzyme CYP2R1 (25-hydroxylase) transforms vitamin D to 25OHD. 25OHD has a long half-life and is usually used to determine vitamin D status in the blood. The second hydroxylation step takes place in the kidneys. In the proximal renal tubule, 1α-hydroxylase (CYP27B1) converts 25OHD into 1,25(OH)_2_D. Active 1,25(OH)_2_D is released into the blood stream and binds to the vitamin D-binding protein. As a lipophilic hormone, 1,25(OH)_2_D can cross the cell membrane and can bind to the VDR present in the cytoplasm and/or nucleus of target cells. The VDR is a ligand-activated transcription factor and regulates gene expression. Over 30 cell types expressing VDR are known, suggesting multiple roles of vitamin D beyond mineral homeostasis [23]. 

The metabolism of vitamin D is strongly regulated by calcium, phosphate, fibroblast growth factor 23 (FGF23), and parathyroid hormone (PTH) levels. FGF23 inhibits 1α-hydroxylase, thereby downregulating 1,25(OH)_2_D production and promoting its catabolism [24]. On the other hand, PTH upregulates CYP27B1 expression in the kidneys, exerting the opposite effect. An increase in ionized blood calcium inhibits PTH secretion in the parathyroid glands, consequently leading to lower 1,25(OH)_2_D production [25]. Hyperphosphatemia also has an inhibitory effect on 1α-hydroxylase activity in the kidneys. When in excess, 1,25(OH)_2_D initiates negative feedback mechanisms by downregulating the expression of the CYP27B1 gene in the kidney, downregulating the expression of the gene encoding PTH in parathyroid glands, and upregulating FGF23 secretion in the skeleton [24,26]. Due to the tight endocrine regulation of renal vitamin D hormone production, circulating 1,25(OH)_2_D levels remain within physiological limits even in the presence of very low 25(OH)D levels associated with severe vitamin D deficiency [27]. This regulation in vitamin D-deficient subjects occurs at the expense of elevated PTH levels, leading to subsequent mobilization of calcium from the skeleton and increased bone turnover, together with potential untoward cardiovascular effects of increased PTH. 

**Figure 1:**
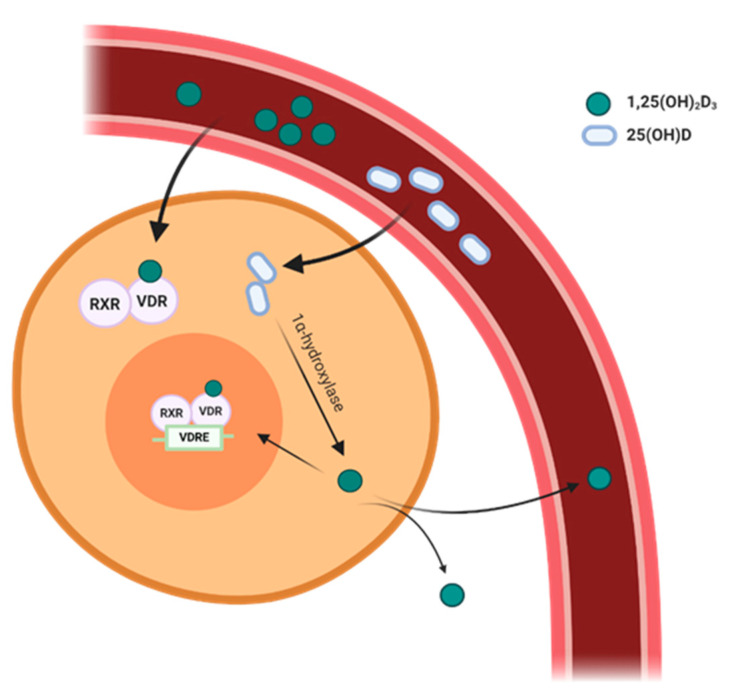
Biological effects of vitamin D signaling in vitamin D receptor (VDR)-expressing target cells are determined by the sum of circulating 1,25(OH)_2_D concentrations in addition to locally produced 1,25(OH)_2_D. The 1,25(OH)_2_D_3_–VDR complex dimerizes with the retinoid X receptor (RXR) and translocates to the nucleus where it binds to vitamin D response elements (VDRE) found in the promoter region of target genes. 25(OH)D originating from the blood stream can be locally converted into 1,25(OH)_2_D in cells expressing 1α-hydroxylase.

Importantly, vitamin D can be activated extra-renally in tissues expressing 1α-hydroxylase. Hence, circulating 25(OH)D also serves as a substrate for local 1,25(OH)_2_D synthesis. Locally produced 1,25(OH)_2_D may have autocrine and paracrine effects in cells that express the VDR. Therefore, the biological effects of vitamin D signaling in VDR-expressing target cells are presumably determined by the sum of circulating 1,25(OH)_2_D concentrations in addition to locally produced 1,25(OH)_2_D (Figure 1). Hence, the blood concentrations of 25(OH)D, routinely used to assess vitamin D status, are not a direct read-out for the activity of vitamin D signaling within target cells.

## 3. Expression of VDR and 1α-Hydroxylase in the Cardiovascular System

The vitamin D receptor was first identified in cardiovascular tissues in low-salt chromatin preparations in normal rat hearts by Walters et al. in 1986 [28]. The demonstration of the presence of a specific receptor for vitamin D in the heart suggested a direct role of vitamin D in maintaining cardiovascular function. In the heart, the VDR is expressed in ventricular cardiomyocytes, as well as in fibroblasts [29,30]. Furthermore, VDR expression was found in cultured bovine aortic endothelial cells and endothelial cells lining the rat aorta [31,32]. This was later confirmed in venular and capillary endothelial cells of human skin biopsies by immunohistochemistry [31]. Moreover, cultured rat cardiomyocytes and cardiac fibroblasts also express 1α-hydroxylase at messenger RNA (mRNA) and protein level [30,31,33], and local production of 1,25(OH)_2_D from labeled 25(OH)D substrate was observed in both endothelial and vascular smooth muscle cells in vitro [33,34]. Additionally, VDR expression was upregulated when cardiomyocytes were subjected to hypertrophic stimuli (endothelin) in vitro and in vivo after treatment with isoproterenol [30]. Therefore, there is solid evidence for both VDR and 1α-hydroxylase expression in the heart and in blood vessels. However, it is less clear what the molecular function of vitamin D signaling in these tissues is, and whether there is an autocrine and/or paracrine regulation of locally produced 1,25(OH)_2_D.

## 4. Hypertension

The observation of the association between high blood pressure and 25(OH)D levels was actually the starting point for considering the involvement of vitamin D in the pathogenesis of cardiovascular disease [35,36]. Hypertension is a major public health burden and a major risk factor for myocardial infarction, heart and kidney failure, and stroke. In most cases, the etiology of hypertension is unknown, leading to patients taking multiple anti-hypertension medications and still having difficulties regulating it. About 20% of the patients suffer from resistant hypertension [37].

Endothelial dysfunction contributes to the development of hypertension. Experimental studies support a role for vitamin D in regulating endothelial function. One of the proposed mechanisms of action is renin–angiotensin–aldosterone (RAAS) activation. It was reported that global VDR knock-out mice have higher blood pressure and develop cardiac hypertrophy due to increased renin expression and subsequent activation of the RAAS [38]. A major limitation of the latter study was that the VDR-null mice were fed a normal diet. VDR-null mice on a normal diet develop severe secondary hyperparathyroidism [39]. Therefore, the increased renin mRNA expression found in VDR-null mice fed the normal diet and the subsequent cardiovascular changes in these animals may be due to secondary hyperparathyroidism [40]. Indeed, global VDR mutant mice fed the so-called rescue diet enriched with calcium, phosphate, and lactose, which normalizes mineral homeostasis in these mice, did not show increased mean arterial pressure or RAAS activation [40]. Rather, Andrukhova et al. found that vitamin D signaling regulates vascular tone, as evidenced by an increase in systolic and pulse pressure, together with altered collagen and elastin content in aged global VDR knock-out animals on the rescue diet, independent of RAAS activation [40]. In line with the changes in pulse pressure, expression of endothelial nitric oxide synthase (eNOS) was reduced by 50% in aortic tissue from VDR global knock-out mice on rescue diet in comparison to wild-type controls [40]. A caveat of studies in global VDR knockout mice is the ubiquitous expression of the VDR, making it difficult to dissect between systemic and tissue-specific effects. However, the findings in global VDR knock-out mice fed the rescue diet were confirmed in a study using mice with specific deletion of the VDR in the endothelium, which do not show changes in mineral metabolism [41]. In the absence of endothelial VDR, acetylcholine-induced aortic relaxation was significantly impaired. Additionally, eNOS mRNA levels were reduced by 60% in the conditional knock-out mice when compared to the controls [41]. Taken together, the data from experimental studies in global and conditional VDR knockout mice support the role of vitamin D signaling in the regulation of vascular tone by upregulating eNOS activity. Chronic treatment of spontaneously hypertensive rats with 1,25(OH)_2_D led to a reduction in reactive oxygen species (ROS) levels and cyclooxygenase-1 (COX-1) mRNA and protein expression [42]. Therefore, another mechanism via which vitamin D may modulate vascular tone is the downregulation of COX-1 in the endothelium, thereby reducing the production of endothelium-derived contracting factors [43]. 

In agreement with the experimental data, a large body of observational studies supports the notion that vitamin D has a protective effect against the development of hypertension. A seasonal variation in blood pressure was recognized, with lower values in the summer and higher in the winter period, when UV light exposure and circulating 25OHD concentrations are lower [35]. In the large NHANES (National Health and Nutrition Examination Survey) III cross-sectional study representative of United States (US) civilians, an inverse relationship between 25(OH)D levels and blood pressure after adjusting for age, sex, ethnicity, and physical activity was shown [44]. However, the effect was attenuated after correcting for body mass index and PTH, suggesting that PTH may mediate most of the association between 25(OH) and blood pressure [45]. A possible limitation of the study was that blood pressure and 25(OH)D levels were measured only at a single time point. In an ancillary study, Judd et al. observed a decrease in the age-related increase in systolic blood pressure in patients with 25(OH)D levels greater than 80 nmol/L [46]. A study with a majority of non-hypertensive subjects found that the age-related increase in systolic blood pressure was lower (0.40 mm Hg/y) in vitamin D-sufficient subjects when compared to vitamin D-deficient and -insufficient participants [46]. In addition, Wang et al. reported that patients with low circulating 25(OH)D are at a higher risk of developing cardiovascular events, including incident hypertension, in comparison to vitamin D-sufficient controls [47].

Forman and coworkers examined the association between hypertension and 25(OH)D levels in the Health Professional Follow-up and Nurses’ Health study [48]. This study included 613 men and 1021 women who did not suffer from hypertension. They found an association between vitamin D deficiency and incident hypertension. One of the major limitations of the study was that the blood pressure measurement was self-reported. However, since the participants were health professionals, the data obtained may be considered reliable. The findings were similar in a Finnish study that assessed 25(OH)D and CVD risk levels at baseline and in a four-year follow up. The association between hypertension and vitamin D deficiency was only significant at baseline, whereas the change in pulse rate over time remained associated with lower 25(OH)D baseline levels in male participants after four years [49]. Kunutsor et al. preformed a meta-analysis of prospective studies that included 283,537 participants [50]. They included data from eight unique prospective cohorts, including 55,816 patients with hypertension. The analysis included patients from the general population with 25(OH)D levels measured at baseline and studies where vitamin D status was assessed based on dietary intake. The results showed that participants in the top third with the highest 25(OH)D levels had a 30% lower risk of hypertension. 

Even though the majority of observational studies reported an inverse association between vitamin D and incidence of hypertension, there are studies that did not observe such an effect. Snijder et al. investigated the relationship among vitamin D, PTH levels, and systolic and diastolic blood pressure in elderly subjects [51]. The latter authors observed no positive effect of vitamin D supplementation but noted that PTH was a potentially modifiable determinant of blood pressure. Similar observations were made in a study investigating the prevalence of metabolic syndrome in patients with low circulating 25(OH)D and high PTH levels [52]. The cohort enrolled over 1000 participants above the age of 40 years. No evidence of an association between low 25(OH)D levels and metabolic syndrome, including hypertension, was found. However, the study suggested that hyperparathyroidism may contribute to the development of metabolic syndrome. 

It is clear that observational studies cannot prove causality. In addition, a fundamental confounder in observational studies could be the fact that subjects with good health may have higher 25(OH)D levels due to higher outdoor activity and subsequently higher sunlight exposure. In order to test a causal relationship, intervention studies investigated the effects of dietary vitamin D supplementation on hypertension. Some of these studies reported beneficial effects of vitamin D on hypertension. Experiments where subjects were exposed to UV radiation recorded a decrease in blood pressure in patients with mild hypertension [53]. Similarly, short-term high doses of vitamin D_3_ (4000 IU) and Ca supplements lowered blood pressure in elderly German women [54]. Forman and coworkers conducted a four-arm controlled trial and reported a 0.2 mm Hg reduction in systolic blood pressure for each 1 mg/mL 25(OH)D. Diastolic pressure remained unchanged [48,55]. In the meta-analysis by Witham et al., which included eight randomized controlled trials, albeit with a low number of participants, a significant reduction of diastolic blood pressure was observed in hypertensive patients (>140 mm Hg) receiving vitamin D supplements [56]. However, another meta-analysis including 16 trials did not reveal a significant protective effect of vitamin D supplementation against hypertension, with the exception of participants with pre-existing cardiometabolic disease [57].

However, in young, normotensive, vitamin D-deficient or -insufficient subjects, dietary vitamin D supplementation increased 25(OH)D levels but had only a negligible effect on blood pressure [58,59,60,61]. In the Daylight trial, which included subjects with low vitamin D status, high doses of vitamin D had no effect on blood pressure [58]. Participants’ age ranged from 18–50, and both normotensive and hypertensive subject were included. The strongest evidence against the beneficial effects of vitamin D supplementation on blood pressure came from the large randomized, double-blind, placebo-controlled trials VIDA and vitamin D treating patients with chronic heart failure (VINDICATE), observing no beneficial effects on systolic or diastolic blood pressure after high-dose vitamin D supplementation [20,62].

Taken together, there is good evidence from experimental studies in genetically engineered mice that ablation of vitamin D signaling is associated with endothelial dysfunction due to a downregulation of eNOS expression. In addition, the majority of observational studies suggests that vitamin D status influences blood pressure and the risk of hypertension. However, the recent large randomized controlled trials provided overwhelming evidence that vitamin D supplementation does not have beneficial effects on blood pressure in vitamin D-sufficient subjects. The potential explanation for this discrepancy may be related to the fact that, due to counter-regulatory mechanisms, the circulating and probably also local 1,25(OH)_2_D concentrations within target cells can be kept within physiological limits in the presence of a large range of circulating 25OHD concentrations, such that additional supplementation may not alter vitamin D signaling in target cells. Nevertheless, based on the experimental data, the vitamin D signaling pathway appears to be important for the regulation of vascular tone. Whether vitamin D analogues could be used as pharmacological agents for the treatment of hypertension is currently unclear.

## 5. Atherosclerosis

Atherosclerosis is caused by an interaction of genetic and environmental factors. Most common factors promoting the development of this condition are hypertension and elevated low-density lipoproteins in the blood. There is some evidence from experimental and clinical studies that vitamin D signaling may modulate the pathogenesis of atherosclerosis. 

Vitamin D signaling may influence the pathophysiology of atherosclerosis through modulation of the inflammatory response by decreasing the expression of TNFα, IL-6, IL-1, and IL-8 in isolated blood monocytes [43,63]. Suppression of IL-6 leads to decreased synthesis of the acute-phase inflammatory C-reactive protein (CRP). CRP serum concentrations are associated with atherosclerosis and serve as a predictor of cardiovascular events [43,64]. Vitamin D deficiency was shown to accelerate progression of coronary artery disease in swine by enhancing nuclear factor-κB (NF-κB) activation, indirectly supporting the anti-inflammatory role of vitamin D [65].

Macrophage-derived foam cell formation is a hallmark of the progression of atherosclerosis [66,67]. Vitamin D was shown to reduce cholesterol accumulation in macrophages and LDL uptake in atheromas [68]. Moreover, it modulates thrombomodulin and tissue factor expression in monocytes, affecting platelet aggregation and thrombogenic activity [69]. In the study by Nakagawa et al., 1,25(OH)_2_D reduced the expression of matrix metalloproteinase (MMP)-2 and MMP-9 in cell culture, thereby possibly preventing plaque destabilization, luminal rupture, and thrombosis [70]. Similarly, foam cell formation in macrophages isolated from hypertensive, diabetic, and obese patients was suppressed by 1,25(OH)_2_D. The proposed mechanism of action involves reduction of low-density lipoprotein uptake [71].

One of major consequences of atherosclerosis is plaque disruption, causing acute obstruction of a coronary artery and, consequently, the development of myocardial infarction (MI). Experimental studies show conflicting evidence regarding the role of vitamin D in the development of MI. Bae and coworkers reported detrimental effects of global VDR deficiency after experimental MI induced by permanent ligation of the left descending coronary artery in global VDR knock-out mice fed a normal diet [72]. By contrast, cardiac function after experimental MI was not different between normocalcemic VDR mutant mice on the rescue diet and wild-type mice [73]. Therefore, the detrimental effects of global VDR deficiency in MI mice may be attributed to hypocalcemia and secondary hyperparathyroidism [73].

Clinical data addressing the role of vitamin D in atherosclerosis are limited. In peripheral arterial disease (PAD), a condition resulting from atherosclerosis and characterized by occlusion of smaller arteries preventing blood flow to the lower extremities, low serum 25(OH)D levels were associated with a higher prevalence after accounting for demographic characteristics, physical activity, and cardiovascular risk factors in NHANES [74]. Similarly, Fahrleitner et al. observed significantly lower serum 25(OH)D levels in patients with PAD in comparison to healthy age-matched control patients [75,76].

The outcome of intervention studies examining the role of vitamin D supplementation on atherosclerosis is diverse. In patients with non-dialysis chronic kidney disease, a high dose of vitamin D positively influenced endothelial function as measured by flow-mediated dilation [77]. Similarly, vitamin D supplementation of 50,000 IU every two weeks for six months led to a significant attenuation of vascular inflammation and an increase in plasma nitric oxide concentration in patients with type 2 diabetes and coronary artery disease [78]. In addition, in patients with high risk of developing chronic diseases like diabetes, supplementation of cholecalciferol or ergocalciferol for four months led to improvements in pulse wave velocity [79]. However, in healthy, elderly people, daily supplementation with 4000 IU for one year induced no changes regarding any cardiovascular risk factors, including arterial stiffness [80]. 

In a double-blinded placebo-controlled trial in MI patients that examined the acute effects of daily vitamin D supplementation on adhesion molecules and proinflammatory cytokines, daily administration of 4000 IU for five days affected some inflammation markers, such as CRP and IL-6, whereas other markers remained unchanged. The major limitations of the latter study were the short duration and the small number of participants. In patients with coronary artery disease, weekly ergocalciferol administration did not result in significant improvements of markers of vascular or endothelial function after 12 weeks [81]. Nonetheless, in subjects with prior history of MI, two high doses (100,000 IU) of cholecalciferol had no effect on blood pressure or cholesterol levels but significantly impacted CRP [82]. In addition, the SYNTAX score was significantly decreased after six months of daily supplementation of calcitriol, indicating that 1,25(OH)_2_D may have a beneficial role in coronary artery disease [83]. By contrast, a monthly bolus supplementation of 100,000 IU of vitamin D over three years had no effect on the incidence of cardiovascular disease, including arteriosclerosis, in the ViDA study [19]. 

In conclusion, there is conflicting evidence from experimental and clinical studies regarding the role of vitamin D signaling in the pathogenesis of atherosclerosis. Based on the well-established role of vitamin D signaling in monocytes/macrophages in many experimental settings [84] and the key role of foam cell formation in the pathogenesis of atherosclerosis, it is conceivable that vitamin D signaling may have beneficial effects in atherosclerosis through an anti-inflammatory action. However, currently, this notion is not substantiated by data from long-term randomized controlled trials. 

## 6. Heart Failure

Left-ventricular hypertrophy (LVH) typically develops as a response to volume or pressure overload, for example, in patients with hypertension or aortic valve stenosis. It is characterized by thickening of the wall and often an increase in chamber size. In pathological hypertrophy, the increase in cardiomyocyte size is accompanied by interstitial fibrosis, cardiac dysfunction, and eventually heart failure. Cardiac fibrosis is of multi-factorial origin and can be either reactive or reparative. Reparative fibrosis occurs in ischemia due to tissue injury and cell death. A scar that predominantly contains collagen I fibers replaces the cells. Reactive fibrosis occurs in the interstitium and around blood vessels without significant loss of cardiomyocytes [85]. The increase of extracellular matrix components in the heart leads to stiffening of the walls and functional impairment eventually leading to heart failure. 

Data from a number of experimental studies support the anti-fibrotic and anti-hypertrophic role of vitamin D, and they propose that vitamin D signaling has a beneficial role in cardiac dysfunction, hypertrophy, and fibrosis [86,87,88,89]. In vitro treatment with 1,25(OH)_2_D resulted in a decrease of profibrotic gene expression and collagen deposition in multipotent mesenchymal stem cells [88]. Furthermore, Chen and coworkers found that specific lack of VDR in cardiomyocytes causes LVH in mice, under normal resting conditions, as well as following a seven-day infusion with isoproterenol, compared to controls [87]. However, the latter authors did not observe changes in interstitial fibrosis. It was suggested that the anti-hypertrophic role of VDR signaling in the heart is based on suppression of the calcineurin/NFAT/MCIP 1 pathway [87]. In addition, in vitro data suggest that vitamin D signaling can improve cardiomyocyte contraction and relaxation [29]. In Dahl salt-sensitive rats, administration of paricalcitol reduced LV mass, posterior wall thickness, and end-diastolic pressure [89].

Epidemiological studies demonstrated that vitamin D deficiency is highly prevalent in subjects suffering from coronary artery disease or heart failure [90,91,92]. Lower 25(OH)D concentrations were independently associated with an increased risk for all-cause mortality and heart failure re-hospitalization in many observational studies [93,94,95]. Furthermore, vitamin D was found to be a predictor of reduced survival in patients with heart failure [90]. In the Framingham heart study, low serum 25(OH)D was associated with a 60% increase in death due to cardiovascular events [96]. A meta-analysis of several observational studies demonstrated a positive association between low 25(OH)D levels and MI, LVH, heart failure, and mortality [94]. In line with this finding, the study by Ameri et al. showed that subjects with intermediate 25(OH)D levels have most favorable LV geometry [97]. Patients with moderate or severe vitamin D deficiency had a significantly higher LV wall thickness and diameter in comparison to the rest of the population studied [97]. This was confirmed in a study consisting of newly diagnosed hypertensive patients with preserved ejection fraction, in whom vitamin D deficiency was associated with higher LV mass and impaired myocardial performance index [98]. However, in a cross-sectional study assessing baseline 25(OH)D levels and left diastolic function, no correlation was found [99]. Moreover, in the PIVUS (Prospective Investigation of Vasculature in Uppsala Seniors) study that included over 870 subjects with no prior cardiovascular disease, no association of LV geometry or function with vitamin D status was established after a five-year period [100]. The Iceland study also failed to find an association between vitamin D status and heart function, but revealed a positive relation between PTH levels and decreased LV function [101]. This goes in line with increasing evidence proposing an association among PTH levels, hypertension, diastolic dysfunction, and hypertrophy [102,103,104,105]. 

The most common cause for mortality in patients suffering from chronic kidney disease is of cardiovascular origin. It was shown that treatment of patients with chronic kidney disease with vitamin D analogues reduces cardiovascular mortality, and it can lead to regression of LVH [106]. In agreement with this finding, low serum 25(OH)D levels were associated with higher LV mass index in an eight-year follow-up in patients with low kidney function [107]. However, after adjusting for PTH levels, no strong relationship could be confirmed. 

Intervention studies provided conflicting evidence. In the RECORD (Randomised Evaluation of Calcium and/OR vitamin D) study, patients receiving 800 IU of cholecalciferol daily were protected against heart failure, but not against MI or stroke [108]. Several other studies showed beneficial effects of vitamin D supplements or its analogues in reducing inflammatory markers [109,110]. By contrast, LV function remained unchanged after administration of 2000 IU vitamin D for nine months [109]. However, another study observed improved ejection fraction in elderly patients with heart failure that received 4000 IU for six months [110]. Such discrepancies may be the consequence of different dosages and time points. It should also be noted that for most of the studies cardiovascular diseases were not the primary endpoints. In the EVITA study (effect of vitamin D on all-cause moratlity in heart failure), 400 patients suffering from heart failure were treated with 4000 IU of vitamin D or placebo for three years. No difference in primary (mortality) or secondary (hospitalization, resuscitation, heart transplantation) end points was observed [111]. The vitamin D treating patients with chronic heart failure (VINDICATE) study comprised 229 participants that received a daily dose of 4000 IU vitamin D for a year. The primary endpoint was a change in 6-min walk distance between baseline and 12 months. Secondary endpoints included changes in LV ejection fraction. No improvements were seen in the primary end point, but vitamin D had a beneficial effect on LV structure and function in these patients. The vitamin D assessment study (ViDA) investigated effects of monthly bolus supplementation of vitamin D on cardiovascular disease, fractures, and cancer. Participants received bolus supplements of 100,000 IU of vitamin D_3_ or placebo for a median of 3.3 years. The primary end point was the number of participants with incident CVD and death. Secondary outcomes were MI, angina, heart failure, hypertension, arrhythmias, arteriosclerosis, stroke, and venous thrombosis. No beneficial effect was found on incidence of cardiovascular disease [19]. The vitamin D and omega 3 trial (VITAL) is a recent double-blinded, randomized, placebo-controlled trial that investigated the influence of high dose vitamin D (2000 IU) and omega 3 fatty-acid supplementation in 25,871 participants. The mean age of the participants was 67.1; they had no history of cardiovascular diseases, and 51% of the subjects were females. The study had a large general population sample, was racially diverse, and included 20% people of color. 25(OH)D levels were measured at baseline and one year following the treatment. The median follow-up was 5.3 years. Major cardiovascular events were primary and secondary end points. Interestingly, the use of vitamin D supplementation neither lowered the incidence of cardiovascular events nor led to a significant difference in any of the secondary cardiovascular end points in comparison to the placebo group [20,21]. A limitation of the VITAL trial was that most subjects were vitamin D-sufficient, and that different results might have occurred if this would have not been the case. However, such a study is not possible in humans due to ethical concerns.

Taken together, despite the evidence from experimental studies that vitamin D signaling has a protective effect against the development of LVH, the outcome of the recent randomized controlled studies clearly shows that vitamin D supplementation of vitamin D-sufficient subjects does not provide any benefit in terms of cardiovascular health in the general population. 

## 7. Conclusions

Data, especially from studies in genetically engineered mice, provided compelling evidence that vitamin D signaling is essential for cardiovascular integrity, especially for the regulation of vascular tone and as an antifibrotic and antihypertrophic signaling pathway in the heart. However, the largely negative results of recent randomized controlled trials with cardiovascular primary and secondary endpoints strongly argue against any beneficial role of vitamin D supplements, at least in vitamin D-sufficient subjects, for cardiovascular health. How can this discrepancy be reconciled? 

Vitamin D is essential for the survival of most vertebrates [112]. Therefore, powerful regulatory systems evolved during evolution that are able to maintain circulating concentrations of the vitamin D hormone, the active principle of the vitamin D system, within narrow limits, despite variations in the circulating concentrations of the precursor molecule 25(OH)D. Much less is known about the regulation of local production of 1,25(OH)_2_D within cardiovascular target cells. Nevertheless, what counts for the activity of vitamin D signaling in a biological sense is the local concentration of 1,25(OH)_2_D within the target cell, regardless of its origin. As outlined above, ablation of the vitamin D signaling pathway in genetically engineered mice revealed an important physiological function of this signaling pathway for cardiovascular health. This represents the extreme end of the spectrum. By contrast, the endocrine systems involved in the regulation of 1,25(OH)_2_D are able to maintain circulating 1,25(OH)_2_D and possibly also intracellular 1,25(OH)_2_D concentrations within physiological limits across a wide range of circulating 25OHD concentrations. Therefore, it makes biological sense that supplementation of vitamin D sufficient subjects will not change the concentration of 1,25(OH)_2_D within target cells and, thus, will not have any biological effect. However, at the lower end of the circulating 25OHD concentration spectrum, the counter-regulatory mechanisms such as increased PTH, possibly in combination with reduced local production of 1,25(OH)_2_D, may result in untoward cardiovascular changes in vitamin D insufficient subjects. Possible strategies to test this hypothesis may be animal models of vitamin D deficiency, better mimicking the condition in humans. Furthermore, it may be conceivable to run randomized controlled trials powered to include sufficient numbers of vitamin D-deficient participants by analyzing baseline vitamin D status only at the end of the study. However, the ethical implications of the latter strategy need to be very carefully considered, and such an approach may not be feasible in humans due to ethical concerns. 

## References

[1] Holick M.F., Biancuzzo R.M., Chen T.C., Klein E.K., Young A., Bibuld D., Reitz R., Salameh W., Ameri A., Tannenbaum A.D. (2008). Vitamin D2 is as effective as vitamin D3 in maintaining circulating concentrations of 25-hydroxyvitamin D. J. Clin. Endocrinol. Metab..

[2] Hammami M.M., Yusuf A. (2017). Differential effects of vitamin D2 and D3 supplements on 25-hydroxyvitamin D level are dose, sex, and time dependent: A randomized controlled trial. BMC Endocr. Disord..

[3] Heaney R.P., Recker R.R., Grote J., Horst R.L., Armas L.A.G. (2011). Vitamin D3 Is More Potent Than Vitamin D2 in Humans. J. Clin. Endocrinol. Metab..

[4] Houghton L.A., Vieth R. (2006). The case against ergocalciferol (vitamin D2) as a vitamin supplement. Am. J. Clin. Nutr..

[5] Shieh A., Chun R.F., Ma C., Witzel S., Meyer B., Rafison B., Swinkels L., Huijs T., Pepkowitz S., Holmquist B. (2016). Effects of High-Dose Vitamin D2 Versus D3 on Total and Free 25-Hydroxyvitamin D and Markers of Calcium Balance. J. Clin. Endocrinol. Metab..

[6] Bikle D.D. (2014). Vitamin D metabolism, mechanism of action, and clinical applications. Chem. Biol..

[7] O’Riordan J.L.H., Bijvoet O.L.M. (2014). Rickets before the discovery of vitamin D. BoneKEy Rep..

[8] Nair R., Maseeh A. (2012). Vitamin D: The “sunshine” vitamin. J. Pharm. Pharm..

[9] Holick M.F., Binkley N.C., Bischoff-Ferrari H.A., Gordon C.M., Hanley D.A., Heaney R.P., Murad M.H., Weaver C.M. (2011). Evaluation, Treatment, and Prevention of Vitamin D Deficiency: An Endocrine Society Clinical Practice Guideline. J. Clin. Endocrinol. Metab..

[10] Manson J.E., Brannon P.M., Rosen C.J., Taylor C.L. (2016). Vitamin D Deficiency—Is There Really a Pandemic?. N. Engl. J. Med..

[11] Scragg R. (1981). Seasonality of Cardiovascular Disease Mortality and the Possible Protective Effect of Ultra-violet Radiation. Int. J. Epidemiol..

[12] Scragg R. (1982). Seasonal variation of mortality in Queensland. Community Health Stud..

[13] Scragg R., Jackson R., Holdaway I.M., Lim T., Beaglehole R. (1990). Myocardial Infarction Is Inversely Associated With Plasma 25-Hydroxyvitamin D3 Levels: A Community-Based Study. Int. J. Epidemiol..

[14] Al Mheid I., Patel R., Murrow J., Morris A., Rahman A., Fike L., Kavtaradze N., Uphoff I., Hooper C., Tangpricha V. (2011). Vitamin D Status Is Associated With Arterial Stiffness and Vascular Dysfunction in Healthy Humans. J. Am. Coll. Cardiol..

[15] Yiu Y.F., Chan Y.H., Yiu K.H., Siu C.W., Li S.W., Wong L.Y., Lee S.W., Tam S., Wong E.W., Cheung B.M. (2011). Vitamin D deficiency is associated with depletion of circulating endothelial progenitor cells and endothelial dysfunction in patients with type 2 diabetes. J. Clin. Endocrinol. Metab..

[16] Lee J.H., Gadi R., Spertus J.A., Tang F., O’Keefe J.H. (2011). Prevalence of vitamin D deficiency in patients with acute myocardial infarction. Am. J. Cardiol..

[17] London G.M., Guérin A.P., Verbeke F.H., Pannier B., Boutouyrie P., Marchais S.J., Mëtivier F. (2007). Mineral Metabolism and Arterial Functions in End-Stage Renal Disease: Potential Role of 25-Hydroxyvitamin D Deficiency. Clin. J. Am. Soc. Nephrol..

[18] Manousaki D., Mokry L.E., Ross S., Goltzman D., Richards J.B. (2016). Mendelian Randomization Studies Do Not Support a Role for Vitamin D in Coronary Artery Disease. Circulation.

[19] Scragg R., Stewart A.W., Waayer D., Lawes C.M.M., Toop L., Sluyter J., Murphy J., Khaw K.-T., Camargo C.A. (2017). Effect of Monthly High-Dose Vitamin D Supplementation on Cardiovascular Disease in the Vitamin D Assessment Study. JAMA Cardiol..

[20] Manson J.E., Cook N.R., Lee I.M., Christen W., Bassuk S.S., Mora S., Gibson H., Gordon D., Copeland T., D’Agostino D. (2019). Vitamin D Supplements and Prevention of Cancer and Cardiovascular Disease. N. Engl. J. Med..

[21] Djoussé L., Cook N.R., Kim E., Bodar V., Walter J., Bubes V., Luttmann-Gibson H., Mora S., Joseph J., Lee I.-M. (2020). Supplementation With Vitamin D and Omega-3 Fatty Acids and Incidence of Heart Failure Hospitalization. Circulation.

[22] Christakos S., Ajibade D.V., Dhawan P., Fechner A.J., Mady L.J. (2010). Vitamin D: Metabolism. Endocrinol. Metab. Clin. N. Am..

[23] Grundmann S.M., Schutkowski A., Schreier B., Rabe S., König B., Gekle M., Stangl G.I. (2019). Vitamin D Receptor Deficiency Does Not Affect Blood Pressure and Heart Function. Front. Physiol..

[24] Martin A., David V., Quarles L.D. (2012). Regulation and function of the FGF23/klotho endocrine pathways. Physiol. Rev..

[25] Bikle D.D., Patzek S., Wang Y. (2018). Physiologic and pathophysiologic roles of extra renal CYP27b1: Case report and review. Bone Rep..

[26] Anderson P.H., May B.K., Morris H.A. (2003). Vitamin D metabolism: New concepts and clinical implications. Clin. Biochem. Rev..

[27] Kennel K.A., Drake M.T., Hurley D.L. (2010). Vitamin D deficiency in adults: When to test and how to treat. Mayo Clin. Proc..

[28] Walters M.R., Wicker D.C., Riggle P.C. (1986). 1,25-Dihydroxyvitamin D3 receptors identified in the rat heart. J. Mol. Cell Cardiol..

[29] Tishkoff D.X., Nibbelink K.A., Holmberg K.H., Dandu L., Simpson R.U. (2008). Functional Vitamin D Receptor (VDR) in the T-Tubules of Cardiac Myocytes: VDR Knockout Cardiomyocyte Contractility. Endocrinology.

[30] Chen S., Glenn D.J., Ni W., Grigsby C.L., Olsen K., Nishimoto M., Law C.S., Gardner D.G. (2008). Expression of the vitamin D receptor is increased in the hypertrophic heart. Hypertension.

[31] Merke J., Milde P., Lewicka S., Hügel U., Klaus G., Mangelsdorf D.J., Haussler M.R., Rauterberg E.W., Ritz E. (1989). Identification and regulation of 1,25-dihydroxyvitamin D3 receptor activity and biosynthesis of 1,25-dihydroxyvitamin D3. Studies in cultured bovine aortic endothelial cells and human dermal capillaries. J. Clin. Investig..

[32] Wong M.S.K., Delansorne R., Man R.Y.K., Vanhoutte P.M. (2008). Vitamin D derivatives acutely reduce endothelium-dependent contractions in the aorta of the spontaneously hypertensive rat. Am. J. Physiol. Heart Circ. Physiol..

[33] Somjen D., Weisman Y., Kohen F., Gayer B., Limor R., Sharon O., Jaccard N., Knoll E., Stern N. (2005). 25-Hydroxyvitamin D3 -1α-Hydroxylase Is Expressed in Human Vascular Smooth Muscle Cells and Is Upregulated by Parathyroid Hormone and Estrogenic Compounds. Circulation.

[34] Zehnder D., Bland R., Chana R.S., Wheeler D.C., Howie A.J., Williams M.C., Stewart P.M., Hewison M. (2002). Synthesis of 1,25-dihydroxyvitamin D(3) by human endothelial cells is regulated by inflammatory cytokines: A novel autocrine determinant of vascular cell adhesion. J. Am. Soc. Nephrol..

[35] Rostand S.G. (1997). Ultraviolet Light May Contribute to Geographic and Racial Blood Pressure Differences. Hypertension.

[36] Fleck A. (1989). Latitude and ischaemic heart disease. Lancet.

[37] Bunker J., Callister W., Chang C.L., Sever P.S. (2011). How common is true resistant hypertension?. J. Hum. Hypertens..

[38] Li Y.C., Kong J., Wei M., Chen Z.F., Liu S.Q., Cao L.P. (2002). 1,25-Dihydroxyvitamin D(3) is a negative endocrine regulator of the renin-angiotensin system. J. Clin. Investig..

[39] Erben R.G., Soegiarto D.W., Weber K., Zeitz U., Lieberherr M., Gniadecki R., Möller G., Adamski J., Balling R. (2002). Deletion of Deoxyribonucleic Acid Binding Domain of the Vitamin D Receptor Abrogates Genomic and Nongenomic Functions of Vitamin D. Mol. Endocrinol..

[40] Andrukhova O., Slavic S., Zeitz U., Riesen S.C., Heppelmann M.S., Ambrisko T.D., Markovic M., Kuebler W.M., Erben R.G. (2014). Vitamin D Is a Regulator of Endothelial Nitric Oxide Synthase and Arterial Stiffness in Mice. Mol. Endocrinol..

[41] Ni W., Watts S.W., Ng M., Chen S., Glenn D.J., Gardner D.G. (2014). Elimination of Vitamin D Receptor in Vascular Endothelial Cells Alters Vascular Function. Hypertension.

[42] Wong M.S.K., Delansorne R., Man R.Y.K., Svenningsen P., Vanhoutte P.M. (2010). Chronic treatment with vitamin D lowers arterial blood pressure and reduces endothelium-dependent contractions in the aorta of the spontaneously hypertensive rat. Am. J. Physiol. Heart Circ. Physiol..

[43] Kassi E., Adamopoulos C., Basdra E.K., Papavassiliou A.G. (2013). Role of Vitamin D in Atherosclerosis. Circulation.

[44] Scragg R., Sowers M., Bell C. (2007). Serum 25-hydroxyvitamin D, Ethnicity, and Blood Pressure in the Third National Health and Nutrition Examination Survey. Am. J. Hypertens..

[45] He J.L., Scragg R.K. (2011). Vitamin D, Parathyroid Hormone, and Blood Pressure in the National Health and Nutrition Examination Surveys. Am. J. Hypertens..

[46] Judd S.E., Nanes M.S., Ziegler T.R., Wilson P.W., Tangpricha V. (2008). Optimal vitamin D status attenuates the age-associated increase in systolic blood pressure in white Americans: Results from the third National Health and Nutrition Examination Survey. Am. J. Clin. Nutr..

[47] Wang T.J., Pencina M.J., Booth S.L., Jacques P.F., Ingelsson E., Lanier K., Benjamin E.J., D’Agostino R.B., Wolf M., Vasan R.S. (2008). Vitamin D Deficiency and Risk of Cardiovascular Disease. Circulation.

[48] Forman J.P., Giovannucci E., Holmes M.D., Bischoff-Ferrari H.A., Tworoger S.S., Willett W.C., Curhan G.C. (2007). Plasma 25-Hydroxyvitamin D Levels and Risk of Incident Hypertension. Hypertension.

[49] Ke L., Graubard B.I., Albanes D., Fraser D.R., Weinstein S.J., Virtamo J., Brock K.E. (2013). Hypertension, Pulse, and Other Cardiovascular Risk Factors and Vitamin D Status in Finnish Men. Am. J. Hypertens..

[50] Kunutsor S.K., Apekey T.A., Steur M. (2013). Vitamin D and risk of future hypertension: Meta-analysis of 283,537 participants. Eur. J. Epidemiol..

[51] Snijder M.B., Lips P., Seidell J.C., Visser M., Deeg D.J.H., Dekker J.M., Van Dam R.M. (2007). Vitamin D status and parathyroid hormone levels in relation to blood pressure: A population-based study in older men and women. J. Int. Med..

[52] Reis J.P., Von Muhlen D., Kritz-Silverstein D., Wingard D.L., Barrett-Connor E. (2007). Vitamin D, Parathyroid Hormone Levels, and the Prevalence of Metabolic Syndrome in Community-Dwelling Older Adults. Diabetes Care.

[53] Krause R., Bühring M., Hopfenmüller W., Holick M.F., Sharma A.M. (1998). Ultraviolet B and blood pressure. Lancet.

[54] Pfeifer M., Begerow B., Minne H.W., Nachtigall D., Hansen C. (2001). Effects of a Short-Term Vitamin D3 and Calcium Supplementation on Blood Pressure and Parathyroid Hormone Levels in Elderly Women1. J. Clin. Endocrinol. Metab..

[55] Forman J.P., Scott J.B., Ng K., Drake B.F., Suarez E.G., Hayden D.L., Bennett G.G., Chandler P.D., Hollis B.W., Emmons K.M. (2013). Effect of Vitamin D Supplementation on Blood Pressure in Blacks. Hypertension.

[56] Witham M.D., Nadir M.A., Struthers A.D. (2009). Effect of vitamin D on blood pressure: A systematic review and meta-analysis. J. Hypertens..

[57] Kunutsor S.K., Burgess S., Munroe P.B., Khan H. (2014). Vitamin D and high blood pressure: Causal association or epiphenomenon?. Eur. J. Epidemiol..

[58] Arora P., Song Y., Dusek J., Plotnikoff G., Sabatine M.S., Cheng S., Valcour A., Swales H., Taylor B., Carney E. (2015). Vitamin D Therapy in Individuals With Prehypertension or Hypertension. Circulation.

[59] Jorde R., Sneve M., Torjesen P., Figenschau Y. (2010). No improvement in cardiovascular risk factors in overweight and obese subjects after supplementation with vitamin D 3 for 1 year. J. Intern. Med..

[60] Nagpal J., Pande J.N., Bhartia A. (2009). A double-blind, randomized, placebo-controlled trial of the short-term effect of vitamin D3 supplementation on insulin sensitivity in apparently healthy, middle-aged, centrally obese men. Diabet. Med..

[61] Salehpour A., Shidfar F., Hosseinpanah F., Vafa M., Razaghi M., Hoshiarrad A., Gohari M. (2012). Vitamin D3 and the risk of CVD in overweight and obese women: A randomised controlled trial. Br. J. Nutr..

[62] Witte K.K., Byrom R., Gierula J., Paton M.F., Jamil H.A., Lowry J.E., Gillott R.G., Barnes S.A., Chumun H., Kearney L.C. (2016). Effects of Vitamin D on Cardiac Function in Patients With Chronic HF: The VINDICATE Study. J. Am. Coll. Cardiol..

[63] Giulietti A., van Etten E., Overbergh L., Stoffels K., Bouillon R., Mathieu C. (2007). Monocytes from type 2 diabetic patients have a pro-inflammatory profile: 1,25-Dihydroxyvitamin D works as anti-inflammatory. Diabetes Res. Clin. Pr..

[64] Shrivastava A.K., Singh H.V., Raizada A., Singh S.K. (2015). C-reactive protein, inflammation and coronary heart disease. Egypt Heart J..

[65] Chen S., Swier V.J., Boosani C.S., Radwan M.M., Agrawal D.K. (2016). Vitamin D Deficiency Accelerates Coronary Artery Disease Progression in Swine. Arterioscler. Thromb. Vasc. Biol..

[66] Sharma G., She Z.-G., Valenta D.T., Stallcup W.B., Smith J.W. (2010). Scavenger Receptor-Mediated Targeting of Macrophage Foam Cells in Atherosclerotic Plaque Using Oligonucleotide-Functionalized Nanoparticles. Nano Life.

[67] Das R., Ganapathy S., Mahabeleshwar G.H., Drumm C., Febbraio M., Jain M.K., Plow E.F. (2013). Macrophage Gene Expression and Foam Cell Formation Are Regulated by Plasminogen. Circulation.

[68] Yin K., You Y., Swier V., Tang L., Radwan M.M., Pandya A.N., Agrawal D.K. (2015). Vitamin D Protects Against Atherosclerosis via Regulation of Cholesterol Efflux and Macrophage Polarization in Hypercholesterolemic Swine. Arterioscler. Thromb. Vasc. Biol..

[69] Koyama T., Shibakura M., Ohsawa M., Kamiyama R., Hirosawa S. (1998). Anticoagulant Effects of 1α,25-Dihydroxyvitamin D3 on Human Myelogenous Leukemia Cells and Monocytes. Blood.

[70] Nakagawa K., Sasaki Y., Kato S., Kubodera N., Okano T. (2005). 22-Oxa-1α,25-dihydroxyvitamin D3 inhibits metastasis and angiogenesis in lung cancer. Carcinogenesis.

[71] Oh J., Weng S., Felton S.K., Bhandare S., Riek A., Butler B., Proctor B.M., Petty M., Chen Z., Schechtman K.B. (2009). 1,25(OH)_2_ Vitamin D Inhibits Foam Cell Formation and Suppresses Macrophage Cholesterol Uptake in Patients With Type 2 Diabetes Mellitus. Circulation.

[72] Bae S., Singh S.S., Yu H., Lee J.Y., Cho B.R., Kang P.M. (2013). Vitamin D signaling pathway plays an important role in the development of heart failure after myocardial infarction. J. Appl. Physiol..

[73] Ford K., Latic N., Slavic S., Zeitz U., Dolezal M., Andrukhov O., Erben R.G., Andrukhova O. (2018). Lack of vitamin D signalling per se does not aggravate cardiac functional impairment induced by myocardial infarction in mice. PLoS ONE.

[74] Melamed M.L., Muntner P., Michos E.D., Uribarri J., Weber C., Sharma J., Raggi P. (2008). Serum 25-Hydroxyvitamin D Levels and the Prevalence of Peripheral Arterial Disease. Arterioscler. Thromb. Vasc. Biol..

[75] Fahrleitner A., Dobnig H., Obernosterer A., Pilger E., Leb G., Weber K., Kudlacek S., Obermayer-Pietsch B.M. (2002). Vitamin D deficiency and secondary hyperparathyroidism are common complications in patients with peripheral arterial disease. J. Gen. Intern. Med..

[76] Fahrleitner-Pammer A., Obernosterer A., Pilger E., Dobnig H., Dimai H.P., Leb G., Kudlacek S., Obermayer-Pietsch B.M. (2005). Hypovitaminosis D, impaired bone turnover and low bone mass are common in patients with peripheral arterial disease. Osteoporos. Int..

[77] Zhang Q.-Y., Jiang C.-M., Sun C., Tang T.-F., Jin B., Cao D.-W., He J.-S., Zhang M. (2015). Hypovitaminosis D is associated with endothelial dysfunction in patients with non-dialysis chronic kidney disease. J. Nephrol..

[78] Farrokhian A., Raygan F., Bahmani F., Talari H.R., Esfandiari R., Esmaillzadeh A., Asemi Z. (2017). Long-Term Vitamin D Supplementation Affects Metabolic Status in Vitamin D–Deficient Type 2 Diabetic Patients with Coronary Artery Disease. J. Nutr..

[79] Forouhi N.G., Menon R.K., Sharp S.J., Mannan N., Timms P.M., Martineau A.R., Rickard A.P., Boucher B.J., Chowdhury T.A., Griffiths C.J. (2016). Effects of vitamin D2 or D3 supplementation on glycaemic control and cardiometabolic risk among people at risk of type 2 diabetes: Results of a randomized double-blind placebo-controlled trial. Diabetes Obes. Metab..

[80] Hin H., Tomson J., Newman C., Kurien R., Lay M., Cox J., Sayer J., Hill M., Emberson J., Armitage J. (2017). Optimum dose of vitamin D for disease prevention in older people: BEST-D trial of vitamin D in primary care. Osteoporos. Int..

[81] Sokol S.I., Srinivas V., Crandall J.P., Kim M., Tellides G., Lebastchi A., Yu Y., Gupta A.K., Alderman M.H. (2012). The effects of vitamin D repletion on endothelial function and inflammation in patients with coronary artery disease. Vasc. Med..

[82] Witham M.D., Dove F.J., Khan F., Lang C.C., Belch J.J.F., Struthers A.D. (2013). Effects of Vitamin D supplementation on markers of vascular function after myocardial infarction—A randomised controlled trial. Int. J. Cardiol..

[83] Wu Z., Wang T., Zhu S., Li L. (2016). Effects of vitamin D supplementation as an adjuvant therapy in coronary artery disease patients. Scand. Cardiovasc. J..

[84] Wöbke T.K., Sorg B.L., Steinhilber D. (2014). Vitamin D in inflammatory diseases. Front. Physiol..

[85] Biernacka A., Frangogiannis N.G. (2011). Aging and Cardiac Fibrosis. Aging Dis..

[86] Qu H., Lin K., Wang H., Wei H., Ji B., Yang Z., Peng C., Xiao X., Deng H. (2017). 1,25(OH)_2_D_3_ improves cardiac dysfunction, hypertrophy, and fibrosis through PARP1/SIRT1/mTOR-related mechanisms in type 1 diabetes. Mol. Nutr. Food Res..

[87] Chen S., Law C.S., Grigsby C.L., Olsen K., Hong T.-T., Zhang Y., Yeghiazarians Y., Gardner D.G. (2011). Cardiomyocyte-specific deletion of the vitamin D receptor gene results in cardiac hypertrophy. Circulation.

[88] Artaza J.N., Norris K.C. (2009). Vitamin D reduces the expression of collagen and key profibrotic factors by inducing an antifibrotic phenotype in mesenchymal multipotent cells. J. Endocrinol..

[89] Bodyak N., Ayus J.C., Achinger S., Shivalingappa V., Ke Q., Chen Y.S., Rigor D.L., Stillman I., Tamez H., Kroeger P.E. (2007). Activated vitamin D attenuates left ventricular abnormalities induced by dietary sodium in Dahl salt-sensitive animals. Proc. Natl. Acad. Sci. USA.

[90] Gotsman I., Shauer A., Zwas D.R., Hellman Y.M., Keren A., Lotan C., Admon D. (2012). Vitamin D deficiency is a predictor of reduced survival in patients with heart failure; vitamin D supplementation improves outcome. Eur. J. Heart Fail..

[91] Kim D.H., Sabour S., Sagar U.N., Adams S., Whellan D.J. (2008). Prevalence of hypovitaminosis D in cardiovascular diseases (from the National Health and Nutrition Examination Survey 2001 to 2004). Am. J. Cardiol..

[92] Zittermann A., Schleithoff S.S., Koerfer R. (2006). Vitamin D insufficiency in congestive heart failure: Why and what to do about it?. Heart Fail. Rev..

[93] Cubbon R.M., Lowry J.E., Drozd M., Hall M., Gierula J., Paton M.F., Byrom R., Kearney L.C., Barth J.H., Kearney M.T. (2019). Vitamin D deficiency is an independent predictor of mortality in patients with chronic heart failure. Eur. J. Nutr..

[94] Brøndum-Jacobsen P., Benn M., Jensen G.B., Nordestgaard B.G. (2012). 25-Hydroxyvitamin D Levels and Risk of Ischemic Heart Disease, Myocardial Infarction, and Early Death. Arterioscler. Thromb. Vasc. Biol..

[95] Melamed M.L., Michos E.D., Post W., Astor B. (2008). 25-hydroxyvitamin D levels and the risk of mortality in the general population. Arch. Intern. Med..

[96] Mahmood S.S., Levy D., Vasan R.S., Wang T.J. (2014). The Framingham Heart Study and the epidemiology of cardiovascular disease: A historical perspective. Lancet.

[97] Ameri P., Canepa M., Milaneschi Y., Spallarossa P., Leoncini G., Giallauria F., Strait J.B., Lakatta E.G., Brunelli C., Murialdo G. (2013). Relationship between vitamin D status and left ventricular geometry in a healthy population: Results from the Baltimore Longitudinal Study of Aging. J. Intern. Med..

[98] Seker T., Gur M., Ucar H., Turkoglu C., Baykan A.O., Özaltun B., Harbalioglu H., Kalkan G.Y., Kaypakli O., Kuloglu O. (2015). Lower serum 25-hydroxyvitamin D level is associated with impaired myocardial performance and left ventricle hypertrophy in newly diagnosed hypertensive patients. Anatol. J. Cardiol..

[99] Pandit A., Mookadam F., Boddu S., Aryal Pandit A., Tandar A., Chaliki H., Cha S., Lee H.R. (2014). Vitamin D levels and left ventricular diastolic function. Open Heart.

[100] Maggio M., De Vita F., Lauretani F., Ceda G.P., Volpi E., Giallauria F., De Cicco G., Cattabiani C., Melhus H., Michaëlsson K. (2014). Vitamin D and Endothelial Vasodilation in Older Individuals: Data From the PIVUS Study. J. Clin. Endocrinol. Metab..

[101] Van Ballegooijen A.J., Visser M., Cotch M.F., Arai A.E., Garcia M., Harris T.B., Launer L.J., Eiríksdóttir G., Gudnason V., Brouwer I.A. (2013). Serum Vitamin D and Parathyroid Hormone in Relation to Cardiac Structure and Function: The ICELAND-MI Substudy of AGES-Reykjavik. J. Clin. Endocrinol. Metab..

[102] Christensson T., Hellström K., Wengle B. (1977). Blood pressure in subjects with hypercalcaemia and primary hyperparathyroidism detected in a health screening programme. Eur. J. Clin. Investig..

[103] Nainby-Luxmoore J.C., Langford H.G., Nelson N.C., Watson R.L., Barnes T.Y. (1982). A case-comparison study of hypertension and hyperparathyroidism. J. Clin. Endocrinol. Metab..

[104] Piovesan A., Molineri N., Casasso F., Emmolo I., Ugliengo G., Cesario F., Borretta G. (1999). Left ventricular hypertrophy in primary hyperparathyroidism. Effects of successful parathyroidectomy. Clin. Endocrinol. (Oxf.).

[105] Almqvist E.G., Bondeson A.G., Bondeson L., Nissborg A., Smedgård P., Svensson S.E. (2002). Cardiac dysfunction in mild primary hyperparathyroidism assessed by radionuclide angiography and echocardiography before and after parathyroidectomy. Surgery.

[106] Shoji T., Shinohara K., Kimoto E., Emoto M., Tahara H., Koyama H., Inaba M., Fukumoto S., Ishimura E., Miki T. (2004). Lower risk for cardiovascular mortality in oral 1 -hydroxy vitamin D3 users in a haemodialysis population. Nephrol. Dial. Transpl..

[107] Van Ballegooijen A.J., Snijder M.B., Visser M., van den Hurk K., Kamp O., Dekker J.M., Nijpels G., Stehouwer C.D., Henry R.M., Paulus W.J. (2012). Vitamin D in relation to myocardial structure and function after eight years of follow-up: The Hoorn study. Ann. Nutr. Metab..

[108] Avenell A., Maclennan G.S., Jenkinson D.J., McPherson G.C., McDonald A.M., Pant P.R., Grant A.M., Campbell M.K., Anderson F.H., Cooper C. (2012). Long-Term Follow-Up for Mortality and Cancer in a Randomized Placebo-Controlled Trial of Vitamin D3and/or Calcium (RECORD Trial). J. Clin. Endocrinol. Metab..

[109] Schleithoff S.S., Zittermann A., Tenderich G., Berthold H.K., Stehle P., Koerfer R. (2006). Vitamin D supplementation improves cytokine profiles in patients with congestive heart failure: A double-blind, randomized, placebo-controlled trial. Am. J. Clin. Nutr..

[110] Dalbeni A., Scaturro G., Degan M., Minuz P., Delva P. (2014). Effects of six months of vitamin D supplementation in patients with heart failure: A randomized double-blind controlled trial. Nutr. Metab. Cardiovasc. Dis..

[111] Zittermann A., Ernst J.B., Prokop S., Fuchs U., Dreier J., Kuhn J., Knabbe C., Birschmann I., Schulz U., Berthold H.K. (2017). Effect of vitamin D on all-cause mortality in heart failure (EVITA): A 3-year randomized clinical trial with 4000 IU vitamin D daily. Eur. Heart J..

[112] Bouillon R., Suda T. (2014). Vitamin D: Calcium and bone homeostasis during evolution. BoneKEy Rep..

